# Supplementary material for: Population genomics of Vibrionaceae isolated from an endangered oasis reveals local adaptation after an environmental perturbation
Source: BMC Genomics. 2020 Jun 22;21:418. doi: 10.1186/s12864-020-06829-y (PMC7306931; doi:10.1186/s12864-020-06829-y)
Supplement: Supplementary file 1 — Additional file 1: Table S1. Nutrient measures across sampled points. Table S2. Ratio of nutrients concentrations. Table S3. RDP classification of partial 16S rRNA. Table S5. General information of the 42 Vibrionaceae genomes and 5 references used in this study. Table S6. Results of the Benchmarking Universal Single-Copy Orthologs (BUSCO) analysis (see methods) using 452 BUSCOS of 721 species of the Gamma-proteobacteria database. Table S9. GO terms enriched at the unique genes of each clade. Figure S1. Phylogenetic reconstruction of 16S RNA sequences. Figure S2. AdaptML analysis. Figure S3. Random analysis of alpha values within each clade. Figure S4. Analysis of structure of Clade II. Figure S5. Frequency of recombination events. Figure S6. Recombination events across a whole genome alignment. Figure S7. UPGMA and membership of the CCB strains. [file 12864_2020_6829_MOESM1_ESM.docx]

**Additional file for:**

**Title:** Population genomics of Vibrionaceae isolated from an endangered oasis reveals local adaptation after an environmental perturbation.

**Authors:**    Mirna Vazquez-Rosas-Landa^1,2^, Gabriel Yaxal Ponce-Soto^1^, Jonás A. Aguirre-Liguori^1^, Shalabh Thakur^3^, Enrique Scheinvar^1^, Josué Barrera-Redondo^1^, Enrique Ibarra-Laclette^2^, David S. Guttman^3,4^, Luis E. Eguiarte^1^ and Valeria Souza^1*^

**Addresses:**    ^1^Departamento de Ecología Evolutiva, Instituto de Ecología, Universidad Nacional Autónoma de México, Ciudad Universitaria, 04510, Ciudad de México, México.

^2^Red de Estudios Moleculares Avanzados, Instituto de Ecología, A.C. – INECOL, Clúster Científico y Tecnológico BioMimic^®^, Carretera antigua a Coatepec 351, El Haya, 91070 Xalapa, Veracruz, México.

^3^Department of Cell and Systems Biology, University of Toronto, Toronto, Ontario, Canada.

^4^Centre for the Analysis of Genome Evolution and Function, University of Toronto, Toronto, Ontario, Canada.

**Table of Contents:**

| **Additional file 1: Table 1. Nutrient measurements.** | 3 |
| --- | --- |
| **Additional file 1: Table 2. Nutrient concentrations ratio.** | 4 |
| **Additional file 1: Table 3. RDP classification of 16S rRNA.** | 5 |
| **Additional file 2: Table 4. Illumina and 454 reads information and assembly processing.** | (Excel sheet) |
| **Additional file 1: Table 5. Genomes general information.** | 18 |
| **Additional file 1: Table 6. Results of BUSCO analysis.** | 21 |
| **Additional file 2: Table 7. Results of recombination on coding genes.** | (Excel sheet) |
| **Additional file 2: Table 8. Metrics of clusters/gene families under selection.** | (Excel sheet) |
| **Additional file 1: Table 9. Genes found within genome regions found with GWAS analysis.** | (Excel sheet) |
| **Additional file 2: Table 10. GO term annotations of unique genes.** | 24 |
| **Additional file 1: Figure 1. Phylogenetic reconstruction of 16S rRNA.** | 25 |
| **Additional file 1: Figure 2. AdaptML ananalysis.** | 26 |
| **Additional file 1: Figure 3. Analysis of alpha values.** | 27 |
| **Additional file 1: Figure 4. Substructure of clade II.** | 28 |
| **Additional file 1: Figure 5. Frequency of recombination events.** | 29 |
| **Additional file 1: Figure 6. Recombination across genome.** | 30 |
| **Additional file 1: Figure 7. Membership of strains.** | 31 |

**Additional file 1: Table 1**. Nutrient measures across sampled points

| Sediments | | | COT | NT | PT |
| --- | --- | --- | --- | --- | --- |
|  |  |  | mg g-1 | mg g-1 | mg g-1 |
| Ponds | Coordinates | |  |  |  |
| PR1 | N 26 52 16.7 | W 102 01 9.9 | 20.300 | 1.669 | 0.079 |
| PR2 | N 26 52 28.3 | W 102 01 18.8 | 35.600 | 3.966 | 0.197 |
| PR3 | N 26 52 8.2 | W 102 01 16.4 | 33.400 | 2.826 | 0.105 |
| PR4 | N 26 52 18.8 | W 102 01 16.7 | 23.300 | 1.723 | 0.093 |
| PR5 | N 26 52 18.1 | W 102 01 17.5 | 12.400 | 1.388 | 0.059 |
| PR6 | N 26 52 17.5 | W 102 01 18.6 | 45.200 | 5.112 | 0.225 |
| PR7 | N 26 52 17.7 | W 102 01 19.1 | 32.600 | 3.151 | 0.145 |
| PR8 | N 26 52 16.2 | W 102 01 18.6 | 30.000 | 3.434 | 0.247 |
| PR9 | N 26 52 15.7 | W 102 01 15.8 | 11.100 | 0.912 | 0.038 |

| Water | | | COT | NT | PT |
| --- | --- | --- | --- | --- | --- |
|  |  |  |  |  |  |
| Ponds | Coordinates | | mg l-1 | mg l-1 | mg l-1 |
| PR1 | N 26 52 16.7 | W 102 01 9.9 | 408.53 | 11.33 | 1.16 |
| PR2 | N 26 52 28.3 | W 102 01 18.8 | 448.73 | 16.16 | 1.38 |
| PR3 | N 26 52 8.2 | W 102 01 16.4 | 480.71 | 10.17 | 0.98 |
| PR4 | N 26 52 18.8 | W 102 01 16.7 | 186.23 | 5.08 | 1.15 |
| PR5 | N 26 52 18.1 | W 102 01 17.5 | 158.38 | 1.63 | 0.90 |
| PR6 | N 26 52 17.5 | W 102 01 18.6 | 499.55 | 11.70 | 1.27 |
| PR7 | N 26 52 17.7 | W 102 01 19.1 | 133.46 | 2.98 | 1.13 |
| PR8 | N 26 52 16.2 | W 102 01 18.6 | 247.09 | 7.42 | 1.10 |
| PR9 | N 26 52 15.7 | W 102 01 15.8 | 109.88 | 2.32 | 1.16 |

**Additional file 1: Table 2.** Ratio of nutrients concentrations.

| Sediment | | | |
| --- | --- | --- | --- |
| Ponds | C:N | C:P | N:P |
| PR1 | 12.16 | 258.41 | 21.25 |
| PR2 | 8.98 | 180.81 | 20.14 |
| PR3 | 11.82 | 316.91 | 26.81 |
| PR4 | 13.53 | 249.71 | 18.46 |
| PR5 | 8.94 | 211.15 | 23.63 |
| PR6 | 8.84 | 200.54 | 22.68 |
| PR7 | 10.35 | 225.25 | 21.77 |
| PR8 | 8.74 | 121.35 | 13.89 |
| PR9 | 12.17 | 289.57 | 23.79 |

| Ponds | C:N | C:P | N:P |
| --- | --- | --- | --- |
| PR1 | 36.05101 | 350.9708 | 9.735395 |
| PR2 | 27.76795 | 324.6961 | 11.6932 |
| PR3 | 47.25816 | 489.5214 | 10.35845 |
| PR4 | 36.63061 | 161.658 | 4.413194 |
| PR5 | 97.04657 | 175.9778 | 1.813333 |
| PR6 | 42.69658 | 393.9669 | 9.227129 |
| PR7 | 44.84543 | 117.8975 | 2.628975 |
| PR8 | 33.30952 | 223.8134 | 6.719203 |
| PR9 | 47.28055 | 94.5611 | 2 |

**Additional file 1: Table 3.** RDP classification of partial 16S rRNA. This table is divide in two; first part correspond to isolates obtained from water (W) environment and second part from sediment (S), indicated by the second column. For each pond and environment, we obtained bacteria isolates, from which we got the partial sequence of the 16S rRNA gene (See methods for more information). Identification of each 16S rRNA sequence was performed with the classifier tool (Wang *et al.,* 2007).

| Number of strains | Environment from isolation. | Pond of isolation | Isolate ID | Phylum | Class | Order | Family | Genus | Confidence |
| --- | --- | --- | --- | --- | --- | --- | --- | --- | --- |
| 1 | W | 1 | PR1A11P | "Proteobacteria" | Gammaproteobacteria | Chromatiales | Chromatiaceae | Rheinheimera | 1 |
| 2 | W | 1 | PR1A15P | "Proteobacteria" | Gammaproteobacteria | Aeromonadales | Aeromonadaceae | Aeromonas | 1 |
| 3 | W | 1 | PR1A18P | "Proteobacteria" | Gammaproteobacteria | Oceanospirillales | Halomonadaceae | Halomonas | 1 |
| 4 | W | 1 | PR1A1P | "Proteobacteria" | Gammaproteobacteria | Chromatiales | Chromatiaceae | Rheinheimera | 1 |
| 5 | W | 1 | PR1A2PO | "Proteobacteria" | Gammaproteobacteria | "Vibrionales" | Vibrionaceae | Photobacterium | 1 |
| 6 | W | 1 | PR1A31T | "Proteobacteria" | Gammaproteobacteria | "Vibrionales" | Vibrionaceae | Listonella | 0.67 |
| 7 | W | 1 | PR1A41T | "Proteobacteria" | Gammaproteobacteria | "Vibrionales" | Vibrionaceae | Vibrio | 0.88 |
| 8 | W | 1 | PR1A4P | "Proteobacteria" | Gammaproteobacteria | "Enterobacteriales" | Enterobacteriaceae | Citrobacter | 0.97 |
| 9 | W | 1 | PR1A60T | "Proteobacteria" | Gammaproteobacteria | "Vibrionales" | Vibrionaceae | Vibrio | 0.81 |
| 10 | W | 1 | PR1A64T | "Proteobacteria" | Gammaproteobacteria | "Vibrionales" | Vibrionaceae | Vibrio | 0.94 |
| 11 | W | 1 | PR1A73T | "Proteobacteria" | Gammaproteobacteria | "Vibrionales" | Vibrionaceae | Vibrio | 0.88 |
| 12 | W | 1 | PR1A8T | "Proteobacteria" | Gammaproteobacteria | "Vibrionales" | Vibrionaceae | Vibrio | 0.88 |
| 13 | W | 2 | PR2A16PO | "Proteobacteria" | Gammaproteobacteria | "Enterobacteriales" | Enterobacteriaceae | Serratia | 1 |
| 14 | W | 2 | PR2A1T | "Proteobacteria" | Gammaproteobacteria | "Vibrionales" | Vibrionaceae | Vibrio | 0.88 |
| 15 | W | 2 | PR2A25T | "Proteobacteria" | Gammaproteobacteria | "Vibrionales" | Vibrionaceae | Vibrio | 0.88 |
| 16 | W | 2 | PR2A27P | "Proteobacteria" | Gammaproteobacteria | "Vibrionales" | Vibrionaceae | Vibrio | 0.88 |
| 17 | W | 2 | PR2A2P | "Proteobacteria" | Gammaproteobacteria | Aeromonadales | Aeromonadaceae | Aeromonas | 1 |
| 18 | W | 2 | PR2A33P | "Proteobacteria" | Gammaproteobacteria | "Enterobacteriales" | Enterobacteriaceae | Yersinia | 0.57 |
| 19 | W | 2 | PR2A34T | "Proteobacteria" | Gammaproteobacteria | "Vibrionales" | Vibrionaceae | Vibrio | 0.88 |
| 20 | W | 2 | PR2A41P | "Proteobacteria" | Gammaproteobacteria | Aeromonadales | Aeromonadaceae | Aeromonas | 1 |
| 21 | W | 2 | PR2A5T | "Proteobacteria" | Gammaproteobacteria | "Enterobacteriales" | Enterobacteriaceae | Citrobacter | 0.97 |
| 22 | W | 2 | PR2A8T | "Proteobacteria" | Gammaproteobacteria | "Vibrionales" | Vibrionaceae | Vibrio | 0.88 |
| 23 | W | 3 | PR3A10P | "Proteobacteria" | Gammaproteobacteria | "Vibrionales" | Vibrionaceae | Vibrio | 0.97 |
| 24 | W | 3 | PR3A14T | "Proteobacteria" | Gammaproteobacteria | "Vibrionales" | Vibrionaceae | Vibrio | 1 |
| 25 | W | 3 | PR3A19P | "Proteobacteria" | Gammaproteobacteria | Oceanospirillales | Oceanospirillaceae | Marinomonas | 1 |
| 26 | W | 3 | PR3A1PO | "Proteobacteria" | Gammaproteobacteria | Pseudomonadales | Moraxellaceae | Acinetobacter | 1 |
| 27 | W | 3 | PR3A28T | "Proteobacteria" | Gammaproteobacteria | "Vibrionales" | Vibrionaceae | Vibrio | 1 |
| 28 | W | 3 | PR3A34P | "Proteobacteria" | Gammaproteobacteria | Oceanospirillales | Oceanospirillaceae | Marinomonas | 1 |
| 29 | W | 3 | PR3A3PO | "Proteobacteria" | Gammaproteobacteria | "Vibrionales" | Vibrionaceae | Vibrio | 1 |
| 30 | W | 3 | PR3A5PO | "Proteobacteria" | Gammaproteobacteria | "Vibrionales" | Vibrionaceae | Vibrio | 1 |
| 31 | W | 3 | PR3A8P | "Proteobacteria" | Gammaproteobacteria | "Enterobacteriales" | Enterobacteriaceae | Serratia | 0.94 |
| 32 | W | 3 | PR3A8T | "Proteobacteria" | Gammaproteobacteria | "Vibrionales" | Vibrionaceae | Vibrio | 1 |
| 33 | W | 4 | PR4A10T | "Proteobacteria" | Gammaproteobacteria | "Vibrionales" | Vibrionaceae | Vibrio | 0.88 |
| 34 | W | 4 | PR4A11PO | "Proteobacteria" | Gammaproteobacteria | Oceanospirillales | Halomonadaceae | Halomonas | 1 |
| 35 | W | 4 | PR4A13P | "Proteobacteria" | Gammaproteobacteria | Aeromonadales | Aeromonadaceae | Aeromonas | 1 |
| 36 | W | 4 | PR4A14PO | "Proteobacteria" | Gammaproteobacteria | Oceanospirillales | Halomonadaceae | Halomonas | 1 |
| 37 | W | 4 | PR4A15PO | "Proteobacteria" | Gammaproteobacteria | Oceanospirillales | Halomonadaceae | Halomonas | 1 |
| 38 | W | 4 | PR4A16PO | "Proteobacteria" | Gammaproteobacteria | Oceanospirillales | Halomonadaceae | Halomonas | 1 |
| 39 | W | 4 | PR4A17PO | "Proteobacteria" | Gammaproteobacteria | Oceanospirillales | Halomonadaceae | Halomonas | 1 |
| 40 | W | 4 | PR4A19P | "Proteobacteria" | Gammaproteobacteria | "Vibrionales" | Vibrionaceae | Vibrio | 0.88 |
| 41 | W | 4 | PR4A1P | "Proteobacteria" | Gammaproteobacteria | "Enterobacteriales" | Enterobacteriaceae | Yersinia | 0.5 |
| 42 | W | 4 | PR4A1PO | "Proteobacteria" | Gammaproteobacteria | "Vibrionales" | Vibrionaceae | Vibrio | 1 |
| 43 | W | 4 | PR4A1T | "Proteobacteria" | Gammaproteobacteria | "Vibrionales" | Vibrionaceae | Vibrio | 0.88 |
| 44 | W | 4 | PR4A23P | "Proteobacteria" | Gammaproteobacteria | "Vibrionales" | Vibrionaceae | Vibrio | 0.88 |
| 45 | W | 4 | PR4A3PO | "Proteobacteria" | Gammaproteobacteria | "Vibrionales" | Vibrionaceae | Vibrio | 1 |
| 46 | W | 4 | PR4A5T | "Proteobacteria" | Gammaproteobacteria | "Vibrionales" | Vibrionaceae | Vibrio | 0.88 |
| 47 | W | 4 | PR4A6T | "Proteobacteria" | Gammaproteobacteria | "Vibrionales" | Vibrionaceae | Vibrio | 0.88 |
| 48 | W | 4 | PR4A7P | "Proteobacteria" | Gammaproteobacteria | Aeromonadales | Aeromonadaceae | Aeromonas | 1 |
| 49 | W | 4 | PR4A7PO | "Proteobacteria" | Gammaproteobacteria | "Vibrionales" | Vibrionaceae | Vibrio | 1 |
| 50 | W | 4 | PR4A8T | "Proteobacteria" | Gammaproteobacteria | "Vibrionales" | Vibrionaceae | Vibrio | 0.88 |
| 51 | W | 5 | PR5A13PO | "Proteobacteria" | Gammaproteobacteria | Oceanospirillales | Halomonadaceae | Halomonas | 1 |
| 52 | W | 5 | PR5A14PO | "Proteobacteria" | Gammaproteobacteria | Oceanospirillales | Halomonadaceae | Salinicola | 1 |
| 53 | W | 5 | PR5A15PO | "Proteobacteria" | Gammaproteobacteria | Oceanospirillales | Halomonadaceae | Salinicola | 1 |
| 54 | W | 5 | PR5A16PO | "Proteobacteria" | Gammaproteobacteria | Oceanospirillales | Halomonadaceae | Halomonas | 1 |
| 55 | W | 5 | PR5A7PO | "Proteobacteria" | Gammaproteobacteria | Oceanospirillales | Halomonadaceae | Halomonas | 1 |
| 56 | W | 5 | PR5A7T | "Proteobacteria" | Gammaproteobacteria | "Vibrionales" | Vibrionaceae | Vibrio | 1 |
| 57 | W | 6 | PR6A1PO | "Proteobacteria" | Gammaproteobacteria | Oceanospirillales | Halomonadaceae | Halomonas | 1 |
| 58 | W | 6 | PR6A28T1 | "Proteobacteria" | Gammaproteobacteria | "Vibrionales" | Vibrionaceae | Vibrio | 1 |
| 59 | W | 6 | PR6A28T2 | "Proteobacteria" | Gammaproteobacteria | "Vibrionales" | Vibrionaceae | Vibrio | 1 |
| 60 | W | 6 | PR6A3PO | "Proteobacteria" | Gammaproteobacteria | Oceanospirillales | Halomonadaceae | Halomonas | 1 |
| 61 | W | 6 | PR6A3T1 | "Proteobacteria" | Gammaproteobacteria | "Vibrionales" | Vibrionaceae | Vibrio | 1 |
| 62 | W | 6 | PR6A5PO | "Proteobacteria" | Gammaproteobacteria | Aeromonadales | Aeromonadaceae | Aeromonas | 1 |
| 63 | W | 6 | PR6A6T2 | "Proteobacteria" | Gammaproteobacteria | "Vibrionales" | Vibrionaceae | Vibrio | 1 |
| 64 | W | 6 | PR6A8P1 | "Proteobacteria" | Gammaproteobacteria | "Vibrionales" | Vibrionaceae | Vibrio | 1 |
| 65 | W | 6 | PR6A8T | "Proteobacteria" | Gammaproteobacteria | "Vibrionales" | Vibrionaceae | Vibrio | 1 |
| 66 | W | 6 | PR6A9PO | "Proteobacteria" | Gammaproteobacteria | Aeromonadales | Aeromonadaceae | Aeromonas | 1 |
| 67 | W | 7 | PR7A15P | "Proteobacteria" | Gammaproteobacteria | Aeromonadales | Aeromonadaceae | Aeromonas | 1 |
| 68 | W | 7 | PR7A16P | "Proteobacteria" | Gammaproteobacteria | Aeromonadales | Aeromonadaceae | Aeromonas | 1 |
| 69 | W | 7 | PR7A18P | "Proteobacteria" | Gammaproteobacteria | Alteromonadales | Shewanellaceae | Shewanella | 1 |
| 70 | W | 7 | PR7A19P | "Proteobacteria" | Gammaproteobacteria | Aeromonadales | Aeromonadaceae | Aeromonas | 1 |
| 71 | W | 8 | PR8A13PO | "Proteobacteria" | Gammaproteobacteria | Aeromonadales | Aeromonadaceae | Aeromonas | 1 |
| 72 | W | 8 | PR8A14PO | "Proteobacteria" | Gammaproteobacteria | Aeromonadales | Aeromonadaceae | Aeromonas | 1 |
| 73 | W | 8 | PR8A21T | "Proteobacteria" | Gammaproteobacteria | "Enterobacteriales" | Enterobacteriaceae | Serratia | 1 |
| 74 | W | 8 | PR8A22P | "Proteobacteria" | Gammaproteobacteria | Aeromonadales | Aeromonadaceae | Aeromonas | 1 |
| 75 | W | 8 | PR8A3P | "Proteobacteria" | Gammaproteobacteria | "Vibrionales" | Vibrionaceae | Vibrio | 1 |
| 76 | W | 8 | PR8A5P | "Proteobacteria" | Gammaproteobacteria | "Enterobacteriales" | Enterobacteriaceae | Serratia | 1 |
| 77 | W | 8 | PR8A6P | "Proteobacteria" | Gammaproteobacteria | "Vibrionales" | Vibrionaceae | Vibrio | 0.88 |
| 78 | W | 8 | PR8A6T | "Proteobacteria" | Gammaproteobacteria | "Vibrionales" | Vibrionaceae | Vibrio | 0.88 |
| 79 | W | 9 | PR9A10T | "Proteobacteria" | Gammaproteobacteria | "Vibrionales" | Vibrionaceae | Vibrio | 0.88 |
| 80 | W | 9 | PR9A1T | "Proteobacteria" | Gammaproteobacteria | "Vibrionales" | Vibrionaceae | Vibrio | 0.88 |
| 81 | W | 9 | PR9A22P | "Proteobacteria" | Gammaproteobacteria | "Enterobacteriales" | Enterobacteriaceae | Yersinia | 0.57 |
| 82 | W | 9 | PR9A5T | "Proteobacteria" | Gammaproteobacteria | "Vibrionales" | Vibrionaceae | Vibrio | 0.88 |
| 83 | W | 9 | PR9A6P | "Proteobacteria" | Gammaproteobacteria | Alteromonadales | Shewanellaceae | Shewanella | 1 |
| 84 | W | 9 | PR9A6T | "Proteobacteria" | Gammaproteobacteria | "Vibrionales" | Vibrionaceae | Vibrio | 0.88 |
| 85 | W | 9 | PR9A9P | "Proteobacteria" | Gammaproteobacteria | Aeromonadales | Aeromonadaceae | Aeromonas | 1 |
| 86 | W | 9 | PR9A9T | "Proteobacteria" | Gammaproteobacteria | "Vibrionales" | Vibrionaceae | Vibrio | 0.88 |
|  |  |  |  |  |  |  |  |  |  |
| 1 | S | 1 | PR1S10PM | "Proteobacteria" | Gammaproteobacteria | "Vibrionales" | Vibrionaceae | Vibrio | 1 |
| 2 | S | 1 | PR1S11PM | "Proteobacteria" | Gammaproteobacteria | "Vibrionales" | Vibrionaceae | Vibrio | 1 |
| 3 | S | 1 | PR1S14PM | "Proteobacteria" | Gammaproteobacteria | "Vibrionales" | Vibrionaceae | Vibrio | 1 |
| 4 | S | 1 | PR1S16PM | "Proteobacteria" | Gammaproteobacteria | "Enterobacteriales" | Enterobacteriaceae | Serratia | 0.94 |
| 5 | S | 1 | PR1S1P | "Proteobacteria" | Gammaproteobacteria | "Vibrionales" | Vibrionaceae | Vibrio | 0.94 |
| 6 | S | 1 | PR1S1T | "Proteobacteria" | Gammaproteobacteria | "Vibrionales" | Vibrionaceae | Vibrio | 0.88 |
| 7 | S | 1 | PR1S2T | "Proteobacteria" | Gammaproteobacteria | "Vibrionales" | Vibrionaceae | Vibrio | 0.97 |
| 8 | S | 1 | PR1S30P | "Proteobacteria" | Gammaproteobacteria | "Vibrionales" | Vibrionaceae | Vibrio | 0.97 |
| 9 | S | 1 | PR1S3P | "Proteobacteria" | Gammaproteobacteria | "Vibrionales" | Vibrionaceae | Vibrio | 0.97 |
| 10 | S | 1 | PR1S3T | "Proteobacteria" | Gammaproteobacteria | "Vibrionales" | Vibrionaceae | Vibrio | 0.88 |
| 11 | S | 1 | PR1S4P | "Proteobacteria" | Gammaproteobacteria | Chromatiales | Chromatiaceae | Rheinheimera | 1 |
| 12 | S | 1 | PR1S4T | "Proteobacteria" | Gammaproteobacteria | "Vibrionales" | Vibrionaceae | Vibrio | 0.88 |
| 13 | S | 1 | PR1S50P | "Proteobacteria" | Gammaproteobacteria | "Vibrionales" | Vibrionaceae | Vibrio | 0.9 |
| 14 | S | 1 | PR1S5P | "Proteobacteria" | Gammaproteobacteria | "Vibrionales" | Vibrionaceae | Vibrio | 0.92 |
| 15 | S | 1 | PR1S5T | "Proteobacteria" | Gammaproteobacteria | "Vibrionales" | Vibrionaceae | Vibrio | 0.9 |
| 16 | S | 1 | PR1S6T | "Proteobacteria" | Gammaproteobacteria | "Vibrionales" | Vibrionaceae | Vibrio | 0.9 |
| 17 | S | 2 | PR2S10T | "Proteobacteria" | Gammaproteobacteria | "Vibrionales" | Vibrionaceae | Vibrio | 0.88 |
| 18 | S | 2 | PR2S12T | "Proteobacteria" | Gammaproteobacteria | "Vibrionales" | Vibrionaceae | Vibrio | 1 |
| 19 | S | 2 | PR2S13PM | "Proteobacteria" | Gammaproteobacteria | "Vibrionales" | Vibrionaceae | Vibrio | 1 |
| 20 | S | 2 | PR2S15PM | "Proteobacteria" | Gammaproteobacteria | "Vibrionales" | Vibrionaceae | Vibrio | 1 |
| 21 | S | 2 | PR2S17PM | "Proteobacteria" | Gammaproteobacteria | "Vibrionales" | Vibrionaceae | Vibrio | 1 |
| 22 | S | 2 | PR2S17T | "Proteobacteria" | Gammaproteobacteria | "Vibrionales" | Vibrionaceae | Vibrio | 0.88 |
| 23 | S | 2 | PR2S1P | "Proteobacteria" | Gammaproteobacteria | "Vibrionales" | Vibrionaceae | Photobacterium | 1 |
| 24 | S | 2 | PR2S1PM | "Proteobacteria" | Gammaproteobacteria | "Vibrionales" | Vibrionaceae | Vibrio | 1 |
| 25 | S | 2 | PR2S21T | "Proteobacteria" | Gammaproteobacteria | "Vibrionales" | Vibrionaceae | Vibrio | 1 |
| 26 | S | 2 | PR2S24PM | "Proteobacteria" | Gammaproteobacteria | "Vibrionales" | Vibrionaceae | Vibrio | 1 |
| 27 | S | 2 | PR2S24PM2 | "Proteobacteria" | Gammaproteobacteria | "Vibrionales" | Vibrionaceae | Vibrio | 1 |
| 28 | S | 2 | PR2S25P | "Proteobacteria" | Gammaproteobacteria | Aeromonadales | Aeromonadaceae | Aeromonas | 1 |
| 29 | S | 2 | PR2S25P1 | "Proteobacteria" | Gammaproteobacteria | "Vibrionales" | Vibrionaceae | Photobacterium | 1 |
| 30 | S | 2 | PR2S2PM | "Proteobacteria" | Gammaproteobacteria | "Vibrionales" | Vibrionaceae | Vibrio | 1 |
| 31 | S | 2 | PR2S30T | "Proteobacteria" | Gammaproteobacteria | "Vibrionales" | Vibrionaceae | Vibrio | 1 |
| 32 | S | 2 | PR2S44P | "Proteobacteria" | Gammaproteobacteria | "Vibrionales" | Vibrionaceae | Photobacterium | 1 |
| 33 | S | 2 | PR2S4T | "Proteobacteria" | Gammaproteobacteria | "Vibrionales" | Vibrionaceae | Vibrio | 1 |
| 34 | S | 2 | PR2S5P | "Proteobacteria" | Gammaproteobacteria | "Vibrionales" | Vibrionaceae | Vibrio | 0.88 |
| 35 | S | 2 | PR2S5PM | "Proteobacteria" | Gammaproteobacteria | "Vibrionales" | Vibrionaceae | Vibrio | 1 |
| 36 | S | 2 | PR2S7P | "Proteobacteria" | Gammaproteobacteria | Aeromonadales | Aeromonadaceae | Aeromonas | 1 |
| 37 | S | 2 | PR2S8PM | "Proteobacteria" | Gammaproteobacteria | "Vibrionales" | Vibrionaceae | Vibrio | 1 |
| 38 | S | 3 | PR3S12P | "Proteobacteria" | Gammaproteobacteria | "Vibrionales" | Vibrionaceae | Vibrio | 0.97 |
| 39 | S | 3 | PR3S12T | "Proteobacteria" | Gammaproteobacteria | "Vibrionales" | Vibrionaceae | Vibrio | 0.88 |
| 40 | S | 3 | PR3S1P | "Proteobacteria" | Gammaproteobacteria | "Vibrionales" | Vibrionaceae | Vibrio | 0.88 |
| 41 | S | 3 | PR3S1T | "Proteobacteria" | Gammaproteobacteria | "Vibrionales" | Vibrionaceae | Vibrio | 0.88 |
| 42 | S | 3 | PR3S1T1 | "Proteobacteria" | Gammaproteobacteria | "Vibrionales" | Vibrionaceae | Vibrio | 0.72 |
| 43 | S | 3 | PR3S34P | "Proteobacteria" | Gammaproteobacteria | "Vibrionales" | Vibrionaceae | Vibrio | 0.88 |
| 44 | S | 3 | PR3S3T | "Proteobacteria" | Gammaproteobacteria | "Vibrionales" | Vibrionaceae | Vibrio | 0.88 |
| 45 | S | 3 | PR3S5T | "Proteobacteria" | Gammaproteobacteria | "Vibrionales" | Vibrionaceae | Vibrio | 0.88 |
| 46 | S | 3 | PR3S6P | "Proteobacteria" | Gammaproteobacteria | "Vibrionales" | Vibrionaceae | Vibrio | 0.97 |
| 47 | S | 3 | PR3S9T | "Proteobacteria" | Gammaproteobacteria | "Vibrionales" | Vibrionaceae | Vibrio | 0.88 |
| 48 | S | 4 | PR4S10P | "Proteobacteria" | Gammaproteobacteria | Aeromonadales | Aeromonadaceae | Aeromonas | 1 |
| 49 | S | 4 | PR4S10T | "Proteobacteria" | Gammaproteobacteria | "Vibrionales" | Vibrionaceae | Vibrio | 0.88 |
| 50 | S | 4 | PR4S1P | "Proteobacteria" | Gammaproteobacteria | "Vibrionales" | Vibrionaceae | Photobacterium | 1 |
| 51 | S | 4 | PR4S1PM | "Proteobacteria" | Gammaproteobacteria | "Enterobacteriales" | Enterobacteriaceae | Serratia | 1 |
| 52 | S | 4 | PR4S1T | "Proteobacteria" | Gammaproteobacteria | "Vibrionales" | Vibrionaceae | Vibrio | 0.88 |
| 53 | S | 4 | PR4S2P | "Proteobacteria" | Gammaproteobacteria | "Vibrionales" | Vibrionaceae | Photobacterium | 1 |
| 54 | S | 4 | PR4S3PM1 | "Proteobacteria" | Gammaproteobacteria | "Enterobacteriales" | Enterobacteriaceae | Serratia | 1 |
| 55 | S | 4 | PR4S3T | "Proteobacteria" | Gammaproteobacteria | "Vibrionales" | Vibrionaceae | Vibrio | 0.88 |
| 56 | S | 4 | PR4S4P | "Proteobacteria" | Gammaproteobacteria | "Vibrionales" | Vibrionaceae | Photobacterium | 1 |
| 57 | S | 4 | PR4S5T | "Proteobacteria" | Gammaproteobacteria | "Vibrionales" | Vibrionaceae | Vibrio | 0.88 |
| 58 | S | 4 | PR4S6T | "Proteobacteria" | Gammaproteobacteria | "Vibrionales" | Vibrionaceae | Vibrio | 0.88 |
| 59 | S | 4 | PR4S8P | "Proteobacteria" | Gammaproteobacteria | Aeromonadales | Aeromonadaceae | Aeromonas | 1 |
| 60 | S | 4 | PR4S8T | "Proteobacteria" | Gammaproteobacteria | "Vibrionales" | Vibrionaceae | Vibrio | 0.88 |
| 61 | S | 5 | PR5S11PM | "Proteobacteria" | Gammaproteobacteria | Oceanospirillales | Halomonadaceae | Halomonas | 1 |
| 62 | S | 5 | PR5S13PM | "Proteobacteria" | Gammaproteobacteria | Oceanospirillales | Halomonadaceae | Halomonas | 1 |
| 63 | S | 5 | PR5S13T | "Proteobacteria" | Gammaproteobacteria | "Vibrionales" | Vibrionaceae | Vibrio | 0.88 |
| 64 | S | 5 | PR5S14PM | "Proteobacteria" | Gammaproteobacteria | "Vibrionales" | Vibrionaceae | Vibrio | 1 |
| 65 | S | 5 | PR5S14T | "Proteobacteria" | Gammaproteobacteria | "Vibrionales" | Vibrionaceae | Listonella | 0.61 |
| 66 | S | 5 | PR5S5P | "Proteobacteria" | Gammaproteobacteria | "Vibrionales" | Vibrionaceae | Vibrio | 0.88 |
| 67 | S | 6 | PR6S12P | "Proteobacteria" | Gammaproteobacteria | "Vibrionales" | Vibrionaceae | Vibrio | 0.88 |
| 68 | S | 6 | PR6S14T2 | "Proteobacteria" | Gammaproteobacteria | "Vibrionales" | Vibrionaceae | Vibrio | 0.88 |
| 69 | S | 6 | PR6S15P | "Proteobacteria" | Gammaproteobacteria | "Vibrionales" | Vibrionaceae | Vibrio | 1 |
| 70 | S | 6 | PR6S15T | "Proteobacteria" | Gammaproteobacteria | "Enterobacteriales" | Enterobacteriaceae | Yersinia | 0.57 |
| 71 | S | 6 | PR6S20T | "Proteobacteria" | Gammaproteobacteria | "Vibrionales" | Vibrionaceae | Vibrio | 1 |
| 72 | S | 6 | PR6S34P | "Proteobacteria" | Gammaproteobacteria | "Vibrionales" | Vibrionaceae | Vibrio | 0.97 |
| 73 | S | 6 | PR6S7T | "Proteobacteria" | Gammaproteobacteria | "Vibrionales" | Vibrionaceae | Vibrio | 1 |
| 74 | S | 7 | PR7S17T | "Proteobacteria" | Gammaproteobacteria | "Vibrionales" | Vibrionaceae | Vibrio | 0.88 |
| 75 | S | 7 | PR7S1P | "Proteobacteria" | Gammaproteobacteria | Aeromonadales | Aeromonadaceae | Aeromonas | 1 |
| 76 | S | 7 | PR7S2PM | "Proteobacteria" | Gammaproteobacteria | Aeromonadales | Aeromonadaceae | Aeromonas | 1 |
| 77 | S | 7 | PR7S3P | "Proteobacteria" | Gammaproteobacteria | Aeromonadales | Aeromonadaceae | Aeromonas | 1 |
| 78 | S | 7 | PR7S4P | "Proteobacteria" | Gammaproteobacteria | Aeromonadales | Aeromonadaceae | Aeromonas | 1 |
| 79 | S | 7 | PR7S5T | "Proteobacteria" | Gammaproteobacteria | "Vibrionales" | Vibrionaceae | Vibrio | 0.88 |
| 80 | S | 7 | PR7S7P | "Proteobacteria" | Gammaproteobacteria | "Vibrionales" | Vibrionaceae | Vibrio | 0.88 |
| 81 | S | 7 | PR7S9P | "Proteobacteria" | Gammaproteobacteria | Alteromonadales | Pseudoalteromonadaceae | Pseudoalteromonas | 1 |
| 82 | S | 8 | PR8S5SAL | "Proteobacteria" | Gammaproteobacteria | Aeromonadales | Aeromonadaceae | Aeromonas | 1 |
| 83 | S | 8 | PR8S6SAL | "Proteobacteria" | Gammaproteobacteria | Oceanospirillales | Halomonadaceae | Halomonas | 1 |
| 84 | S | 8 | PR8S8SAL | "Proteobacteria" | Gammaproteobacteria | Oceanospirillales | Halomonadaceae | Halomonas | 1 |
| 85 | S | 8 | PR8S9SAL | "Proteobacteria" | Gammaproteobacteria | "Vibrionales" | Vibrionaceae | Vibrio | 0.88 |
| 86 | S | 9 | PR9S11P | "Proteobacteria" | Gammaproteobacteria | Aeromonadales | Aeromonadaceae | Aeromonas | 1 |
| 87 | S | 9 | PR9S4P | "Proteobacteria" | Gammaproteobacteria | Aeromonadales | Aeromonadaceae | Aeromonas | 1 |
| 88 | S | 9 | PR9S7T | "Proteobacteria" | Gammaproteobacteria | "Vibrionales" | Vibrionaceae | Vibrio | 0.83 |

**Additional file 1: Table 5.** General information of the 42 Vibrionaceae genomes and 5 references used in this study.

| Clade | Strain | | Genome size (bp) | % GC | CDSs | BioProject/Seq Accession | BioSample | Isolation |
| --- | --- | --- | --- | --- | --- | --- | --- | --- |
|  |  |  |  |  |  |  |  |  |
| *Vibrio* II | *V. anguillarum* | 755 | 4,052,047 | 44.4 | 3,656 | PRJNA224116 | SAMN02603689 | Coho salmon (*Oncorhynchus kisutch*) |
| *Vibrio* II | *Vibrio sp.* | V01_P9A10T6 | 3,756,599 | 44.7 | 3,464 | PRJNA361510 | SAMN06234507 | CCB Los Hundidos |
| *Vibrio* II | *Vibrio sp.* | V02_P2A34T133 | 3,695,193 | 44.9 | 3,367 | PRJNA361510 | SAMN06251330 | CCB Los Hundidos |
| *Vibrio* II | *Vibrio sp.* | V03_P4A6T147 | 3,474,820 | 45.1 | 3,168 | PRJNA361511 | SAMN06251399 | CCB Los Hundidos |
| *Vibrio* II | *Vibrio sp.* | V04_P4A5T148 | 3,921,253 | 44.6 | 3,464 | PRJNA361511 | SAMN06251400 | CCB Los Hundidos |
| *Vibrio* II | *Vibrio sp.* | V05_P4A8T149 | 3,722,593 | 44.8 | 3,462 | PRJNA361511 | SAMN06251401 | CCB Los Hundidos |
| *Vibrio* II | *Vibrio sp.* | V06_P1A73T115 | 3,571,545 | 45 | 3,274 | PRJNA361511 | SAMN06251402 | CCB Los Hundidos |
| *Vibrio* II | *Vibrio sp.* | V07_P2A8T137 | 3,624,703 | 45 | 3,284 | PRJNA361511 | SAMN06251403 | CCB Los Hundidos |
| *Vibrio* II | *Vibrio sp.* | V08_P9A1T1 | 3,650,269 | 44.9 | 3,302 | PRJNA361511 | SAMN06251404 | CCB Los Hundidos |
| *Vibrio* II | *Vibrio sp.* | V09_P4A23P171 | 3,936,426 | 44.5 | 3,736 | PRJNA361511 | SAMN06251405 | CCB Los Hundidos |
| *Vibrio* II | *Vibrio sp.* | V10_P2A27P122 | 3,803,588 | 44.7 | 3,502 | PRJNA361511 | SAMN06251406 | CCB Los Hundidos |
| *Vibrio* II | *Vibrio sp.* | V11_P1A41T118 | 3,624,696 | 44.9 | 3,368 | PRJNA361511 | SAMN06251407 | CCB Los Hundidos |
| *Vibrio* II | *Vibrio sp.* | V12_P9A6T4 | 3,838,079 | 44.7 | 3,622 | PRJNA361511 | SAMN06251408 | CCB Los Hundidos |
| *Vibrio* II | *Vibrio sp.* | V14_P6S14T42 | 3,849,935 | 44.7 | 3,653 | PRJNA361511 | SAMN06251409 | CCB Los Hundidos |
| *Vibrio* II | *Vibrio sp.* | V15_P4S5T153 | 5,092,048 | 43.2 | 5,004 | PRJNA361511 | SAMN06251410 | CCB Los Hundidos |
| *Vibrio* II | *Vibrio sp.* | V17_P4S1T151 | 4,753,379 | 43.5 | 4,705 | PRJNA361511 | SAMN06251411 | CCB Los Hundidos |
| *Vibrio* II | *Vibrio sp.* | V18_P1S4T112 | 3,846,979 | 44.6 | 3,622 | PRJNA361511 | SAMN06251412 | CCB Los Hundidos |
| *Vibrio* II | *Vibrio sp.* | V19_P1S1T109 | 3,777,969 | 44.8 | 3,470 | PRJNA361511 | SAMN06251413 | CCB Los Hundidos |
| *Vibrio* II | *Vibrio sp.* | V20_P4S3T152 | 3,902,904 | 44.9 | 3,671 | PRJNA361511 | SAMN06251414 | CCB Los Hundidos |
| *Vibrio* II | *Vibrio sp.* | V22_P2S10T140 | 3,735,804 | 44.9 | 3,441 | PRJNA361511 | SAMN06251415 | CCB Los Hundidos |
| *Vibrio* II | *Vibrio sp.* | V23_P3S9T160 | 3,892,924 | 44.5 | 3,698 | PRJNA361511 | SAMN06251416 | CCB Los Hundidos |
| *Vibrio* II | *Vibrio sp.* | V24_P1S3T111 | 3,821,490 | 44.6 | 3,559 | PRJNA361511 | SAMN06251417 | CCB Los Hundidos |
| *Vibrio* II | *Vibrio sp.* | V25_P4S6T154 | 3,927,806 | 44.9 | 3,696 | PRJNA361511 | SAMN06251418 | CCB Los Hundidos |
| *Vibrio* III | *V. metschnikovii* | CIP 69.14 | 3,815,101 | 44.1 | 3,186 | PRJNA224116 | SAMN02393820 | Type strain |
| *Vibrio* III | *Vibrio sp.* | V31_P5A7T61 | 3,689,628 | 44.1 | 3,376 | PRJNA361511 | SAMN06251424 | CCB Los Hundidos |
| *Vibrio* III | *Vibrio sp.* | V32_P6A28T40 | 3,646,568 | 44.2 | 3,337 | PRJNA361511 | SAMN06251425 | CCB Los Hundidos |
| *Vibrio* III | *Vibrio sp.* | V33_P6A3T137 | 3,610,172 | 44.4 | 3,333 | PRJNA361511 | SAMN06251426 | CCB Los Hundidos |
| *Vibrio* III | *Vibrio sp.* | V34_P3A8T189 | 3,680,368 | 44.4 | 3,414 | PRJNA361511 | SAMN06251427 | CCB Los Hundidos |
| *Vibrio* III | *Vibrio sp.* | V40_P2S30T141 | 3,492,560 | 44.6 | 3,173 | PRJNA361511 | SAMN06251432 | CCB Los Hundidos |
|  |  |  |  |  |  |  |  |  |
| *Vibrio* IV | *Vibrio sp.* | V26_P1S5P106 | 3,121,437 | 43.9 | 2,887 | PRJNA361511 | SAMN06251419 | CCB Los Hundidos |
| *Vibrio* IV | *Vibrio sp.* | V27_P1S3P104 | 3,526,383 | 43.4 | 3,297 | PRJNA361511 | SAMN06251420 | CCB Los Hundidos |
| *Vibrio* IV | *Vibrio sp.* | V28_P6S34P95 | 3,503,965 | 43.5 | 3,244 | PRJNA361511 | SAMN06251421 | CCB Los Hundidos |
| *Vibrio* IV | *Vibrio sp.* | V29_P1S30P107 | 3,292,482 | 43.7 | 3,053 | PRJNA361511 | SAMN06251422 | CCB Los Hundidos |
| *Vibrio* IV | *Vibrio sp.* | V30_P3S12P165 | 3,524,720 | 43.4 | 3,266 | PRJNA361511 | SAMN06251423 | CCB Los Hundidos |
| Photobacterium | *Photobacterium sp.* | P44_P4S2P179 | 4,572,902 | 51.2 | 4,389 | PRJNA361511 | SAMN06251436 | CCB Los Hundidos |
| Photobacterium | *Photobacterium sp.* | P45_P2S44P127 | 4,546,445 | 51.3 | 4,368 | PRJNA361511 | SAMN06251437 | CCB Los Hundidos |
| Photobacterium | *Photobacterium sp.* | P46_P4S1P180 | 4,566,757 | 51.3 | 4,406 | PRJNA361511 | SAMN06251438 | CCB Los Hundidos |
| *Vibrio* V | *Vibrio sp.* | V36_P2S2PM302 | 4,900,770 | 50.1 | 4,673 | PRJNA361511 | SAMN06251428 | CCB Los Hundidos |
| *Vibrio* V | *Vibrio sp.* | V37_P2S8PM304 | 4,825,922 | 50.3 | 4,602 | PRJNA361511 | SAMN06251429 | CCB Los Hundidos |
| *Vibrio* V | *Vibrio sp.* | V38_P2S17PM301 | 4,945,276 | 50 | 4,746 | PRJNA361511 | SAMN06251430 | CCB Los Hundidos |
| *Vibrio* V | *Vibrio sp.* | V39_P1S14PM300 | 4,653,839 | 50.4 | 4,386 | PRJNA361511 | SAMN06251431 | CCB Los Hundidos |
| *Vibrio* VI | *V. parahaemolyticus* | BB220p | 5,103,524 | 45.3 | 4,442 | [PRJNA224116](https://www.ncbi.nlm.nih.gov/bioproject/PRJNA224116) | [SAMN02604302](https://www.ncbi.nlm.nih.gov/biosample/SAMN02604302) | Bangladesh environmental isolate |
| *Vibrio* VI | *V. alginolyticus* | NBRC 15630 | 5,146,637 | 44.6 | 4,457 | PRJNA224116 | [SAMN02603463](https://www.ncbi.nlm.nih.gov/biosample/SAMN02603463) | Spoiled horse mackerel |
| *Vibrio* VI | *Vibrio sp.* | V41_P2S12T139 | 3,613,116 | 45.6 | 3,320 | PRJNA361511 | SAMN06251433 | CCB Los Hundidos |
| *Vibrio* VI | *Vibrio sp.* | V42_P2S4T144 | 4,776,336 | 45.1 | 4,383 | PRJNA361511 | SAMN06251434 | CCB Los Hundidos |
| *Vibrio* VI | *Vibrio sp.* | V43_P6S15P86 | 4,846,002 | 45 | 4,468 | PRJNA361511 | SAMN06251435 | CCB Los Hundidos |
| *Vibrio* | *V. furnissii* | NCTC 11218 | 4,937,540 | 50.6 | 4,434 | [PRJNA224116](https://www.ncbi.nlm.nih.gov/bioproject/PRJNA224116) | [SAMN02603955](https://www.ncbi.nlm.nih.gov/biosample/SAMN02603955) | Estuarine environment |

**Additional file 1: Table 6.** Results of the Benchmarking Universal Single-Copy Orthologs (BUSCO) analysis (see methods) using 452 BUSCOS of 721 species of the Gamma-proteobacteria database.

| **Clade** | **Strain** | | **BUSCO** | | | | |
| --- | --- | --- | --- | --- | --- | --- | --- |
|  |  |  | **Complete BUSCOs** | **Complete and single-copy BUSCOs** | **Complete and duplicated BUSCOs** | **Fragmented BUSCOs** | **Missing BUSCOs** |
|  |  |  |  |  |  |  |  |
| *Vibrio* II | *V. anguillarum* | 755 | 444 | 443 | 1 | 6 | 2 |
| *Vibrio* II | *Vibrio sp.* | V01_P9A10T6 | 444 | 444 | 0 | 3 | 5 |
| *Vibrio* II | *Vibrio sp.* | V02_P2A34T133 | 442 | 442 | 0 | 2 | 8 |
| *Vibrio* II | *Vibrio sp.* | V03_P4A6T147 | 430 | 429 | 1 | 5 | 17 |
| *Vibrio* II | *Vibrio sp.* | V04_P4A5T148 | 450 | 450 | 0 | 1 | 1 |
| *Vibrio* II | *Vibrio sp.* | V05_P4A8T149 | 435 | 435 | 0 | 6 | 11 |
| *Vibrio* II | *Vibrio sp.* | V06_P1A73T115 | 441 | 441 | 0 | 2 | 9 |
| *Vibrio* II | *Vibrio sp.* | V07_P2A8T137 | 441 | 441 | 0 | 3 | 8 |
| *Vibrio* II | *Vibrio sp.* | V08_P9A1T1 | 441 | 441 | 0 | 5 | 6 |
| *Vibrio* II | *Vibrio sp.* | V09_P4A23P171 | 451 | 451 | 0 | 1 | 0 |
| *Vibrio* II | *Vibrio sp.* | V10_P2A27P122 | 443 | 443 | 0 | 5 | 4 |
| *Vibrio* II | *Vibrio sp.* | V11_P1A41T118 | 439 | 438 | 1 | 4 | 9 |
| *Vibrio* II | *Vibrio sp.* | V12_P9A6T4 | 449 | 449 | 0 | 1 | 2 |
| *Vibrio* II | *Vibrio sp.* | V14_P6S14T42 | 451 | 451 | 0 | 1 | 0 |
| *Vibrio* II | *Vibrio sp.* | V15_P4S5T153 | 451 | 448 | 3 | 1 | 0 |
| *Vibrio* II | *Vibrio sp.* | V17_P4S1T151 | 442 | 440 | 2 | 4 | 6 |
| *Vibrio* II | *Vibrio sp.* | V18_P1S4T112 | 451 | 451 | 0 | 1 | 0 |
| *Vibrio* II | *Vibrio sp.* | V19_P1S1T109 | 435 | 435 | 0 | 7 | 10 |
| *Vibrio* II | *Vibrio sp.* | V20_P4S3T152 | 448 | 447 | 1 | 1 | 3 |
| *Vibrio* II | *Vibrio sp.* | V22_P2S10T140 | 445 | 445 | 0 | 2 | 5 |
| *Vibrio* II | *Vibrio sp.* | V23_P3S9T160 | 448 | 446 | 2 | 1 | 3 |
| *Vibrio* II | *Vibrio sp.* | V24_P1S3T111 | 450 | 450 | 0 | 1 | 1 |
| *Vibrio* II | *Vibrio sp.* | V25_P4S6T154 | 448 | 447 | 1 | 1 | 3 |
|  |  |  |  |  |  |  |  |
| *Vibrio* III | *V. metschnikovii* | CIP 69.14 | 421 | 421 | 0 | 19 | 12 |
| *Vibrio* III | *Vibrio sp.* | V31_P5A7T61 | 450 | 449 | 1 | 2 | 0 |
| *Vibrio* III | *Vibrio sp.* | V32_P6A28T40 | 450 | 449 | 1 | 2 | 0 |
| *Vibrio* III | *Vibrio sp.* | V33_P6A3T137 | 447 | 446 | 1 | 2 | 3 |
| *Vibrio* III | *Vibrio sp.* | V34_P3A8T189 | 449 | 448 | 1 | 2 | 1 |
| *Vibrio* III | *Vibrio sp.* | V40_P2S30T141 | 440 | 439 | 1 | 5 | 7 |
|  |  |  |  |  |  |  |  |
| *Vibrio* IV | *Vibrio sp.* | V26_P1S5P106 | 408 | 408 | 0 | 7 | 37 |
| *Vibrio* IV | *Vibrio sp.* | V27_P1S3P104 | 442 | 442 | 0 | 5 | 5 |
| *Vibrio* IV | *Vibrio sp.* | V28_P6S34P95 | 448 | 448 | 0 | 2 | 2 |
| *Vibrio* IV | *Vibrio sp.* | V29_P1S30P107 | 433 | 433 | 0 | 4 | 15 |
| *Vibrio* IV | *Vibrio sp.* | V30_P3S12P165 | 450 | 450 | 0 | 2 | 0 |
|  |  |  |  |  |  |  |  |
| Photobacterium | *Photobacterium sp.* | P44_P4S2P179 | 450 | 449 | 1 | 1 | 1 |
| Photobacterium | *Photobacterium sp.* | P45_P2S44P127 | 445 | 445 | 0 | 3 | 4 |
| Photobacterium | *Photobacterium sp.* | P46_P4S1P180 | 443 | 443 | 0 | 2 | 7 |
|  |  |  |  |  |  |  |  |
| *Vibrio* V | *Vibrio sp.* | V36_P2S2PM302 | 451 | 445 | 6 | 1 | 0 |
| *Vibrio* V | *Vibrio sp.* | V37_P2S8PM304 | 448 | 443 | 5 | 3 | 1 |
| *Vibrio* V | *Vibrio sp.* | V38_P2S17PM301 | 452 | 446 | 6 | 0 | 0 |
| *Vibrio* V | *Vibrio sp.* | V39_P1S14PM300 | 432 | 426 | 6 | 5 | 15 |
|  |  |  |  |  |  |  |  |
| *Vibrio* VI | *V. parahaemolyticus* | BB220p | 438 | 434 | 4 | 4 | 10 |
| *Vibrio* VI | *V. alginolyticus* | NBRC 15630 | 449 | 445 | 4 | 3 | 0 |
| *Vibrio* VI | *Vibrio sp.* | V41_P2S12T139 | 346 | 346 | 0 | 18 | 88 |
| *Vibrio* VI | *Vibrio sp.* | V42_P2S4T144 | 421 | 417 | 4 | 6 | 25 |
| *Vibrio* VI | *Vibrio sp.* | V43_P6S15P86 | 441 | 438 | 3 | 5 | 6 |
|  |  |  |  |  |  |  |  |
| *Vibrio* | *V. furnissii* | NCTC 11218 | 432 | 423 | 9 | 14 | 6 |
|  |  |  |  |  |  |  |  |

**Additional file 1: Table 9.** GO terms enriched at the unique genes of each clade.

|  | GO.ID | Term | Annotated | Significant | Expected | Fisher test with Bonferroni correction |
| --- | --- | --- | --- | --- | --- | --- |
| Clade I | GO:0030153 | bacteriocin immunity | 4 | 3 | 0.04 | 0.0075576 |
| Clade II | GO:0016998 | cell wall macromolecule catabolic process... | 78 | 5 | 0.13 | 0.00038544 |
| Clade II | GO:0009253 | peptidoglycan catabolic process | 124 | 5 | 0.21 | 0.0038544 |
| Clade II | GO:0015891 | siderophore transport | 136 | 5 | 0.23 | 0.0061028 |
| Clade III | GO:0009103 | lipopolysaccharide biosynthetic process | 208 | 5 | 0.12 | 0.00022498 |
| Clade IV | GO:0006810 | transport | 16621 | 57 | 31.07 | 0.0013668 |
| Clade IV | GO:0002101 | tRNA wobble cytosine modification | 41 | 4 | 0.08 | 0.0017688 |
| Clade IV | GO:0051391 | tRNA acetylation | 41 | 4 | 0.08 | 0.0017688 |
| Clade IV | GO:0000160 | phosphorelay signal transduction system | 3099 | 20 | 5.79 | 0.002412 |
| Clade IV | GO:0032328 | alanine transport | 121 | 5 | 0.23 | 0.0057888 |
| Clade IV | GO:0007165 | signal transduction | 5325 | 34 | 9.95 | 0.038592 |
| Clade V | GO:0002101 | tRNA wobble cytosine modification | 41 | 4 | 0.04 | 0.0001608 |
| Clade V | GO:0051391 | tRNA acetylation | 41 | 4 | 0.04 | 0.0001608 |
| Clade V | GO:0055085 | transmembrane transport | 5369 | 20 | 5.5 | 0.002412 |
| Clade V | GO:0016310 | phosphorylation | 2230 | 12 | 2.28 | 0.0045024 |


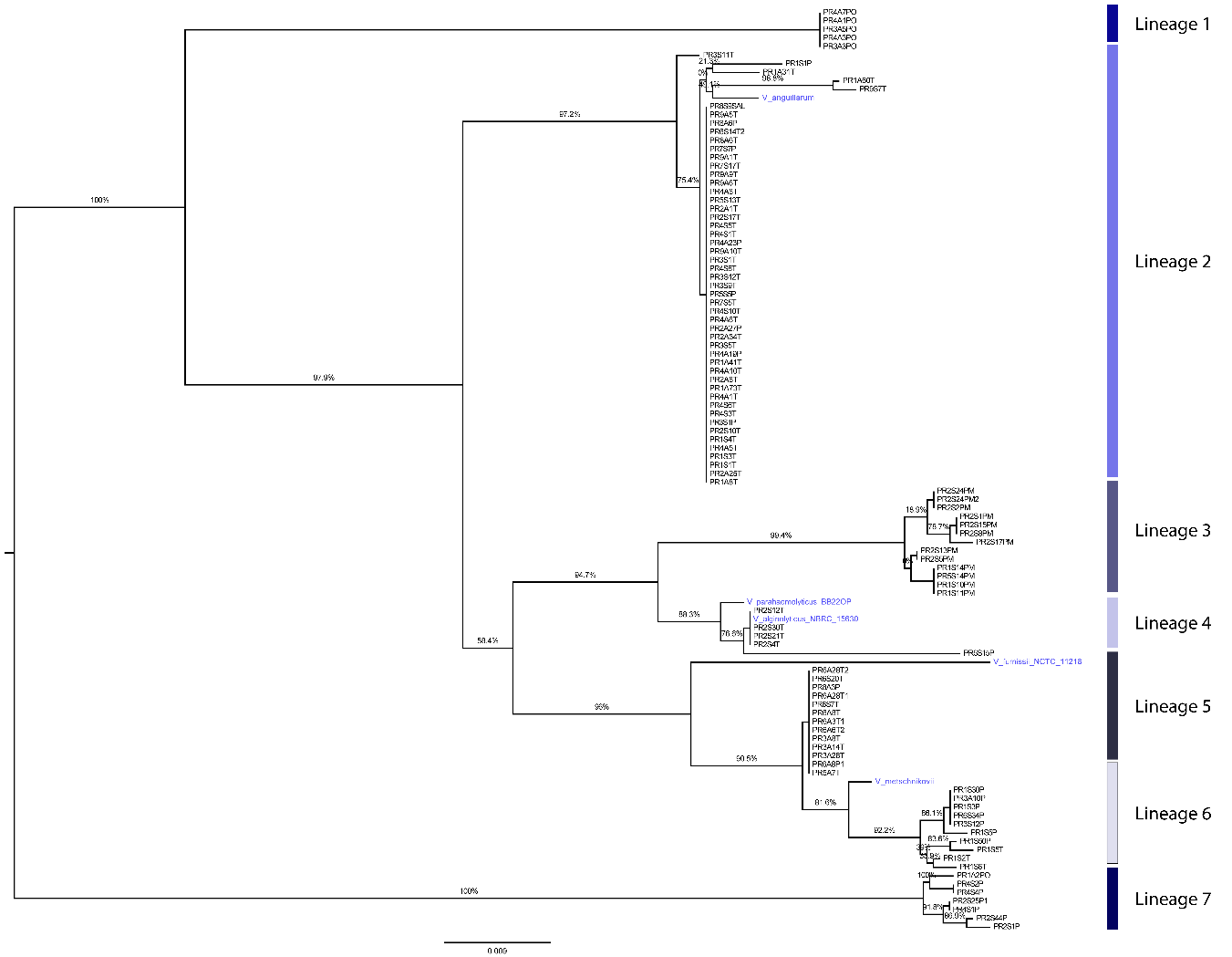


**Additional file 1: Figure 1.** Phylogenetic reconstruction of 16S RNA sequences. A maximum likelihood method was employed over the first 700 bps of the 16S rRNA gene. Reference strains are shown in blue, as well seven lineages are highlighted with different colors. Strains for genome sequencing were selected from lineages, 2, 3, 4, 5, 6 and 7.


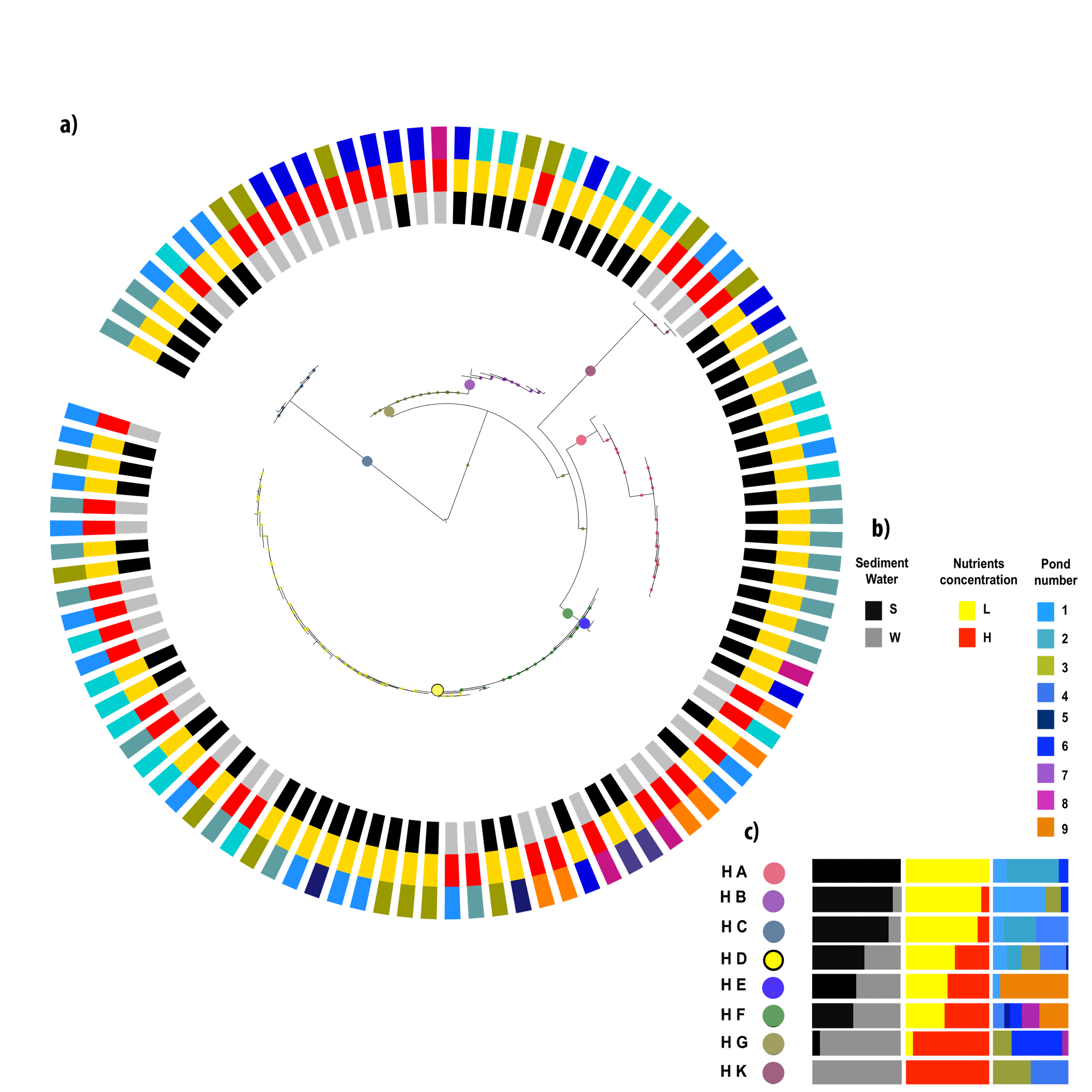


**Additional file 1: Figure 2.** AdaptML analysis. Maximum likelihood phylogeny of partial 16S rRNA gene with *Halomonas* as outgroup. a) Shows the grouping of phylogroups given the environment from where they came from, colored dots within phylogeny indicates the projected habitat to which each phylogroup belong. b) Shows the color key for each environmental label. c) Shows the projected habitats and its specific content.


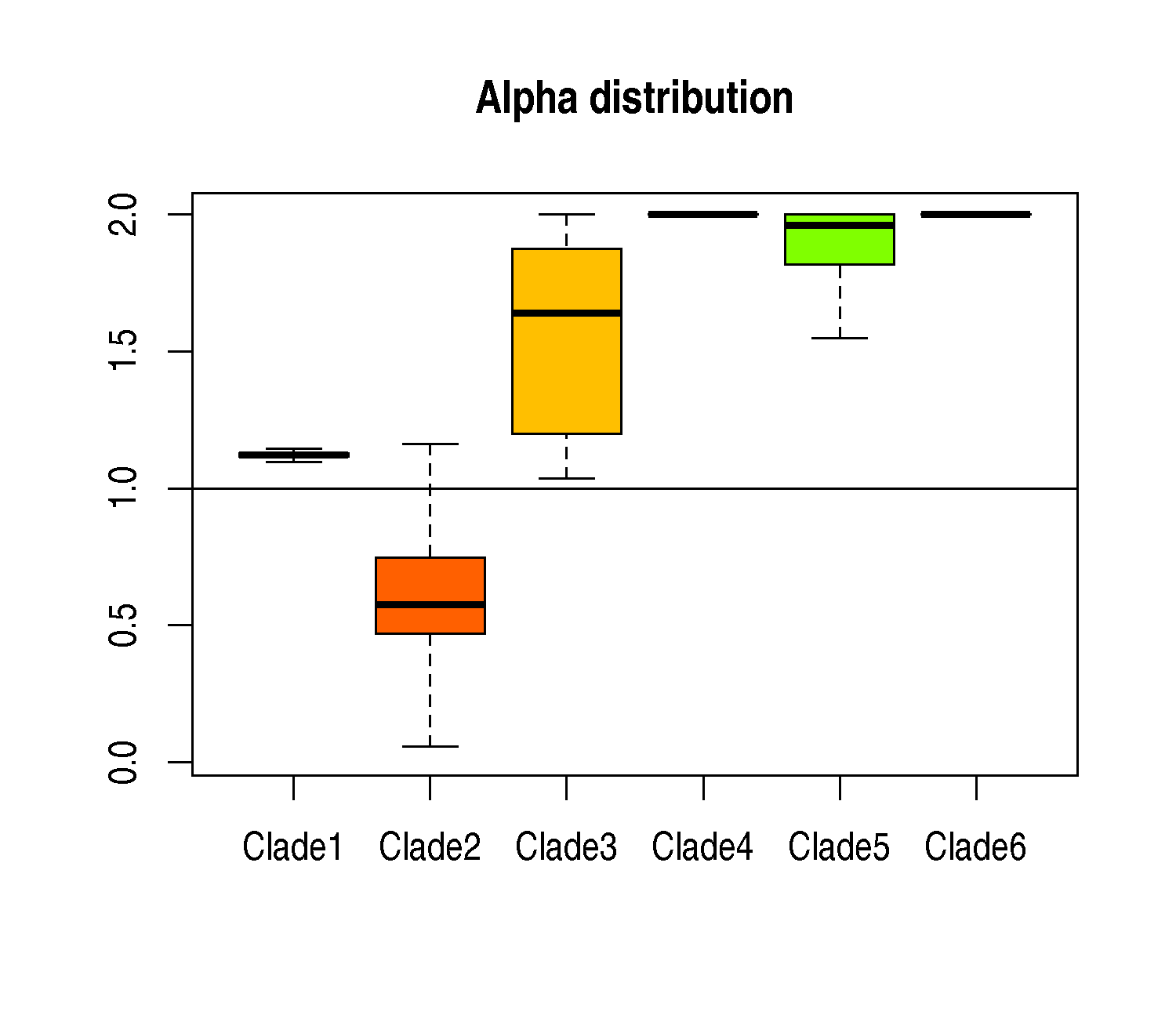


**Additional file 1: Figure 3**. Random analysis of alpha values within each clade. The alpha values of each clade were calculated using three genomes, iterating this process 1000 types and changing randomly the three genomes each round of calculation. The “X” axis corresponds to each clade and “Y” axis to alpha values.


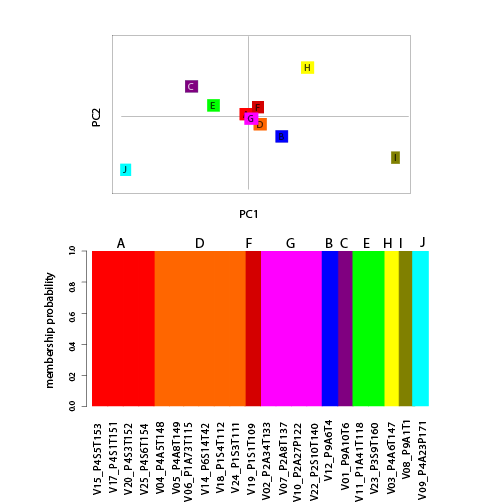


**Additional file 1: Figure 4.** Analysis of structure of Clade II. The discriminant analysis of subgroups of Clade II displayed ten genetic groups (A-J), the strain composition of this genetic groups can be appreciated in the membership probability analysis below. The Sub-clades A, D and G were used for further analysis.


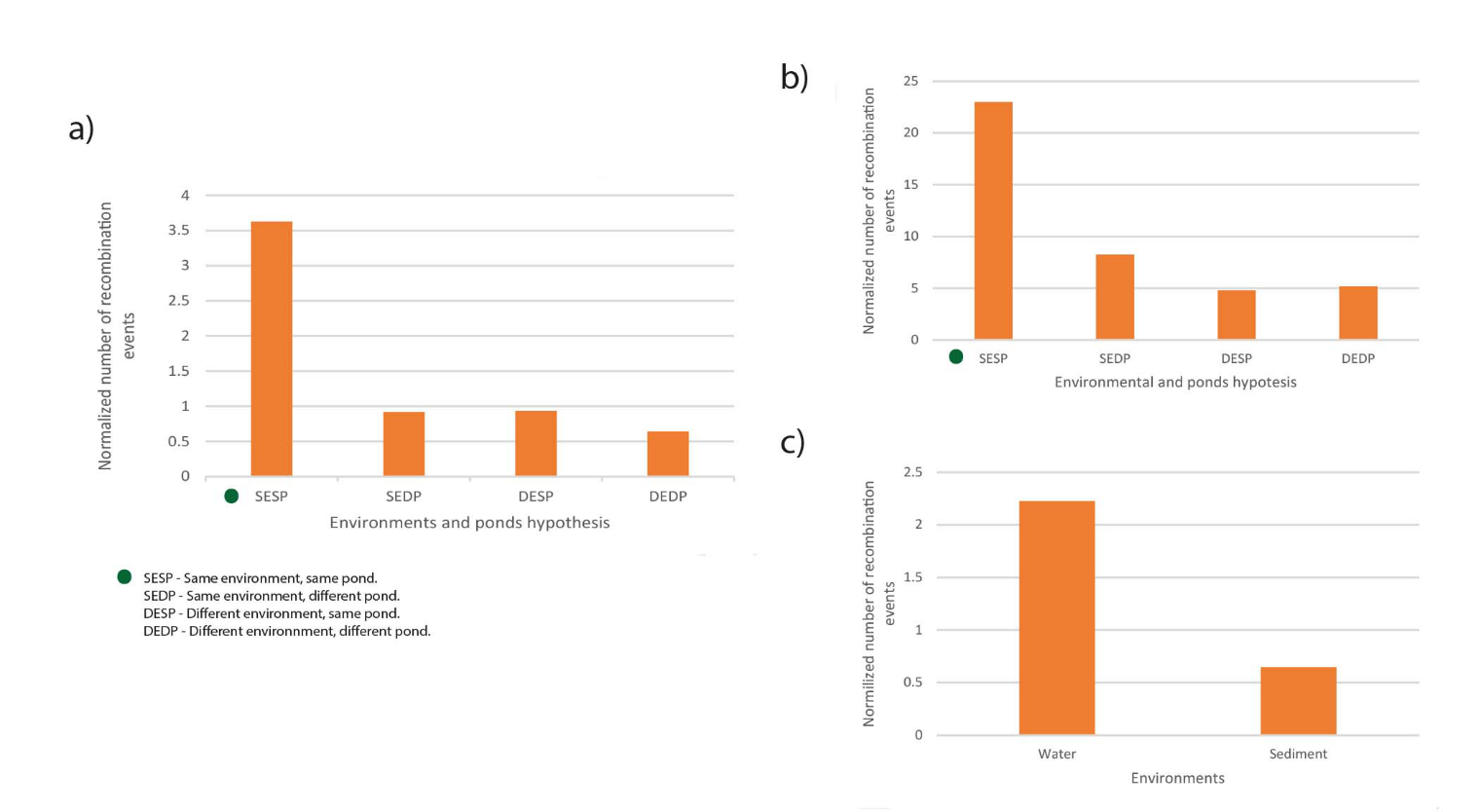


**Additional file 1: Figure 5.** Frequency of recombination events. a) Shows frequency of recombination events of all Vibrionaceae. b) Shows frequency of recombination events based of Clade 2. c) Shows recombination events in terms of the origin of samples.


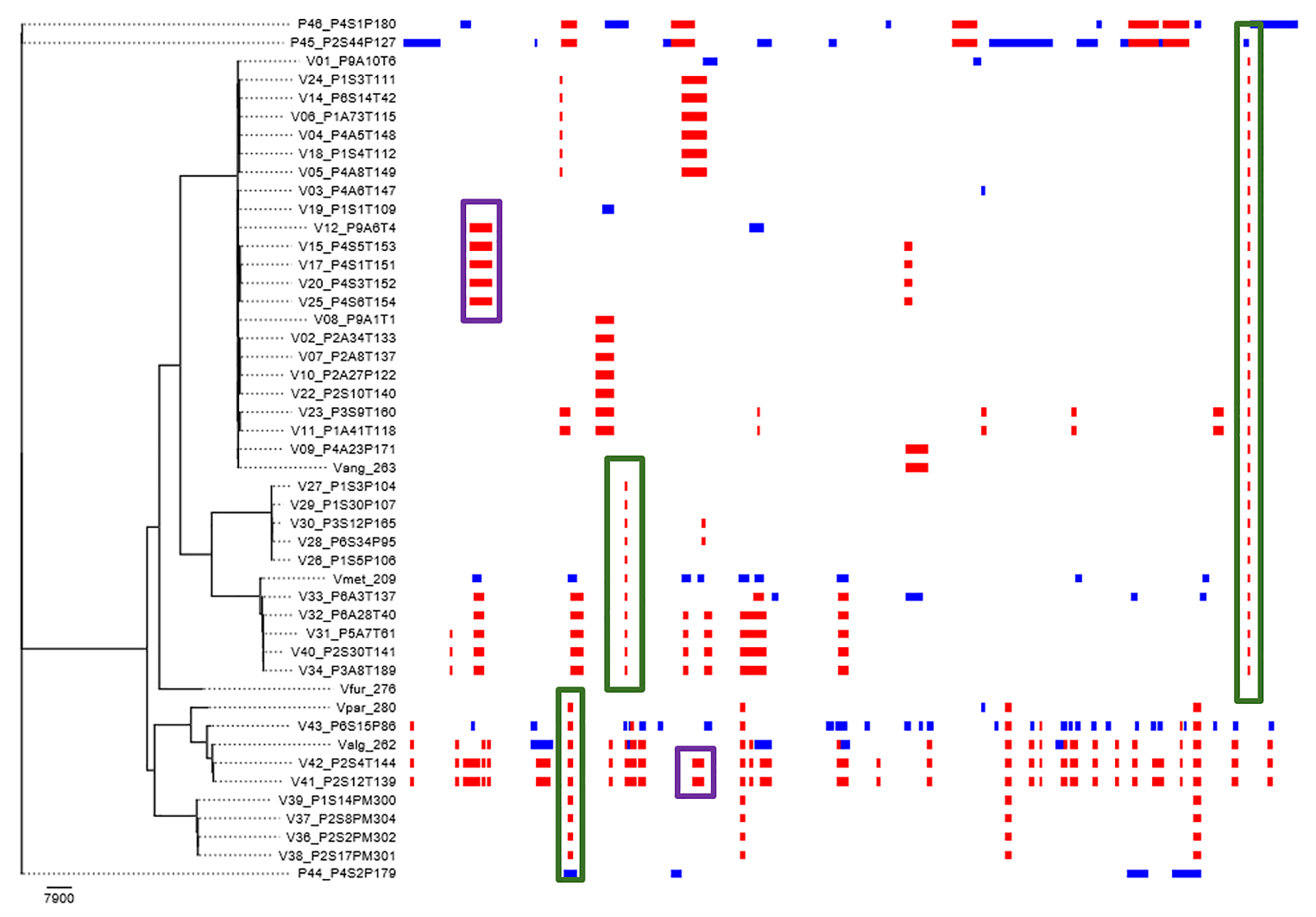


**Additional file 1: Figure 6.** Recombination events across a whole genome alignment. Red blocks represent recombination events inside the alignment, while blue blocks represent points of recombination which origin was probably outside the alignment. Green boxed indicate sites that are shared with references and purple boxes indicate sites that had signals of recombination only within CCB strains.


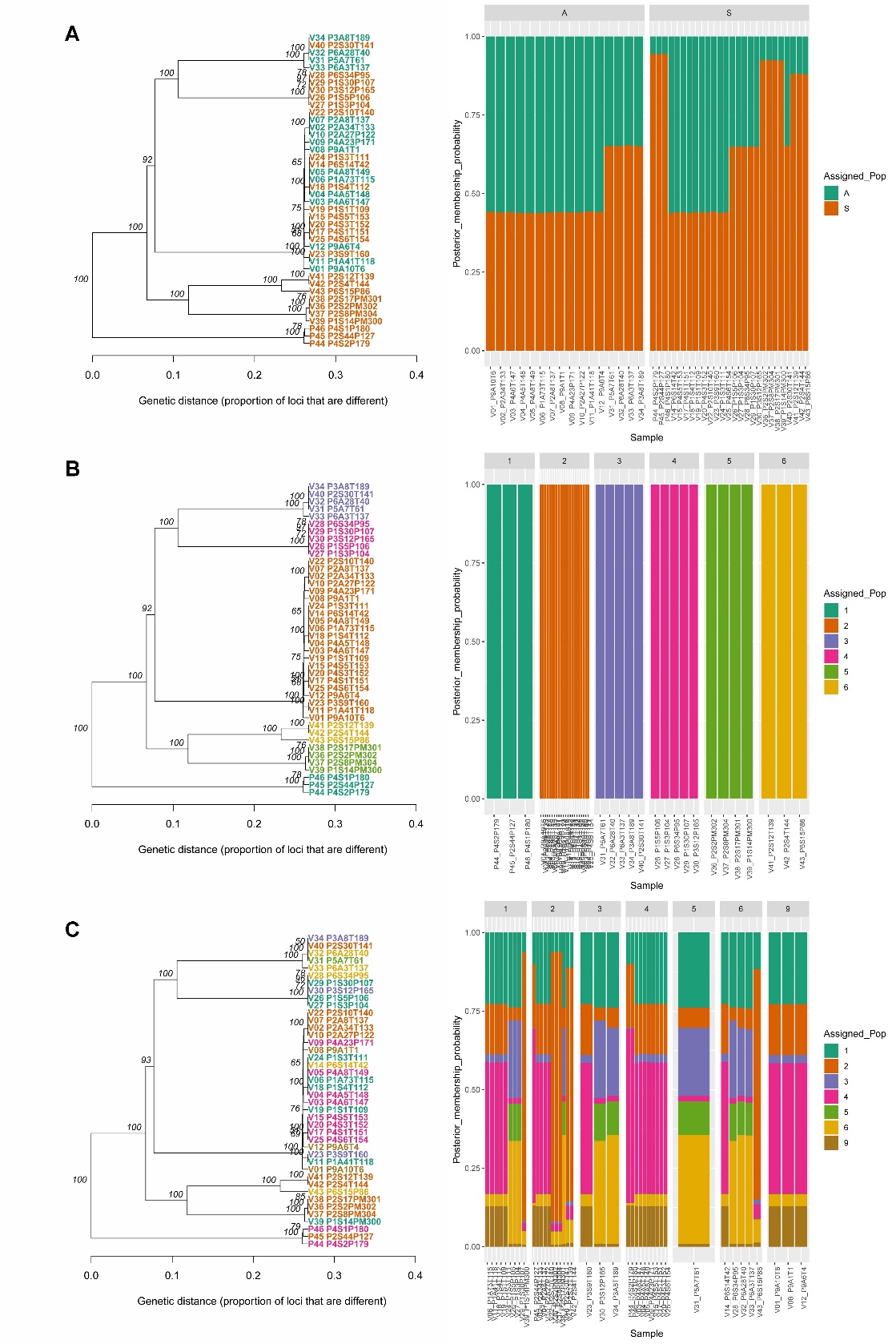


**Additional file 1: Figure 7.** UPGMA and membership of the CCB strains. a) Clustering of the analyze strains according to their isolation environment. b) Clustering of the analyzed strains according to the predicted clade. c) Clustering of the analyzed strains according to their isolation pond.
